# Facile Solvent-Free Preparation of Antioxidant Idebenone-Loaded Nanoparticles for Efficient Wound Healing

**DOI:** 10.3390/pharmaceutics14030521

**Published:** 2022-02-26

**Authors:** HeeSeon Yang, Sohyeon Yu, Jisu Kim, KumJu Baek, Young-Ran Lee, Hye Sun Lee, Won Il Choi, Daekyung Sung

**Affiliations:** 1 Center for Bio-Healthcare Materials, Bio-Convergence Materials R&D Division, Korea Institute of Ceramic Engineering and Technology, 202 Osongsaengmyeong 1-ro, Osong-eup, Heungdeok-gu, Cheongju 28160, Korea; happyenffl@naver.com (H.Y.); wer2807@hanyang.ac.kr (S.Y.); jisukim6650@yonsei.ac.kr (J.K.); oioers@naver.com (K.B.); yrlee@kicet.re.kr (Y.-R.L.); hslee@kicet.re.kr (H.S.L.); 2 Department of Chemical Engineering, Hanyang University, 222 Wangsimni-ro, Seongdong-gu, Seoul 04763, Korea; 3 Department of Chemical and Biomolecular Engineering, Yonsei University, 50 Yonsei-ro, Seodaemun-gu, Seoul 03722, Korea

**Keywords:** idebenone, PEG-40 hydrogenated castor oil, nanoparticle, solubilization, antioxidant activity, wound healing

## Abstract

The excessive production of reactive oxygen species (ROS) causes harmful effects, including biomolecular damage and inflammation. ROS due to ultraviolet rays, blue light, and fine dust harm the skin, causing urban-related aging. Therefore, a strong antioxidant that relieves oxidative stress in the skin and removes ROS is required. Idebenone (IB) is a powerful antioxidant but is poorly soluble and thus has low solubility in water, resulting in low bioavailability. In this study, IB-loaded nanoparticles (IB@NPs) were synthesized by loading IB without an organic solvent into nanoparticles that can provide high loading efficiency and stability for solubilization. Indeed, the synthesized IB@NPs exhibited long-term stability through dynamic light scattering, methylene blue staining, and redispersion assays, and IB@NPs prepared with a 5 wt% IB loading content were found to be optimal. The antioxidant activity of IB@NPs evaluated using the 2,2-diphenyl-1-picrylhydrazyl (DPPH) assay was significantly higher than that of unloaded IB. In addition, IB@NPs showed excellent biocompatibility, inhibited oxidative damage to mouse NIH-3T3 fibroblasts, and reduced intracellular ROS generation according to an in vitro DPPH antioxidant assay. Most notably, IB@NPs significantly promoted wound healing in vitro, as demonstrated by scratch assays. Therefore, as carriers with excellent stability, IB@NPs have potential cosmetic and pharmaceutical applications.

## 1. Introduction

Reactive oxygen species (ROS), including hydrogen peroxide (H_2_O_2_), hydroxyl radicals (OH^-^), and superoxide radicals (O_2_^-^), act as cell proliferation, improvement, and redox messengers for apoptosis [1,2]. However, excessive ROS production causes damage to biomolecules within the cell and induces harmful processes such as inflammation, namely by inflicting oxidative stress [3,4]. ROS are generated on the skin due to ultraviolet rays, blue light, and fine dust that are widely present in modern society, causing urban-related aging and skin cancers [5,6,7,8,9]. Therefore, stable antioxidants that can relieve excessive oxidative stress in the skin and remove ROS are required [10,11,12].

Idebenone (IB), a powerful lipophilic antioxidant, is a synthetic short-chain benzoquinone that is used in the pharmaceutical and cosmetic industries [13,14,15]. Similar to coenzyme Q10, IB can protect the mitochondria from lipid peroxidation and ROS-induced damage [16,17,18]. IB has also been used clinically as a topical treatment for various ROS-mediated diseases [12,19,20], with effects in preventing apoptosis, facilitating cell differentiation, and preventing skin damage and aging, which can reduce inflammation and increase wound-healing activity by minimizing free radicals [18,21,22,23]. However, the greatest disadvantage of IB is that it is poorly soluble; accordingly, its solubility in water is low, resulting in low bioavailability [22,24]. Nanocapsule techniques have been used to improve the bioavailability and solubility of lipophilic drugs, including IB [22]. For example, previous studies encapsulated fat-soluble IB using liposomes, lipid-based nanoparticles, and solid-lipid nanoparticles to improve its bioavailability [14,25,26]. However, its loading efficiency and physical stability are still very low [27]; therefore, a suitable nanocarrier of IB is sought to improve both the loading efficiency and stability. In addition, most IB loading methods reported to date involve the use of organic solvents such as ethanol, tetrahydrofuran, and chloroform, which are known to maximize the solubilization of poorly soluble, physiologically active substances [13,14]. However, using such solvents has disadvantages, including high cost, low biocompatibility, and low eco-friendliness.

**Figure 1:**
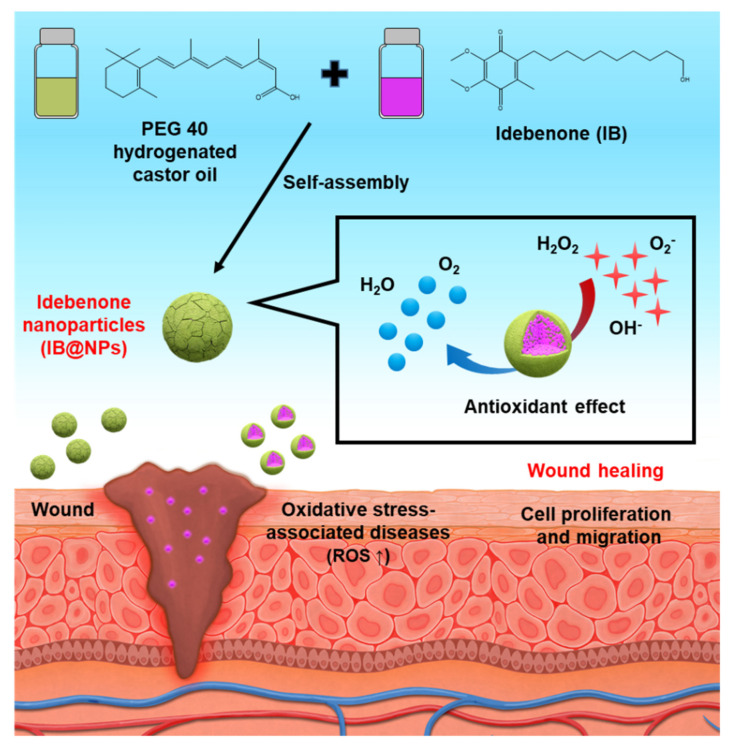
Schematic illustration of idebenone-loaded nanoparticles (IB@NPs), including the preparation of IB@NPs and strong antioxidant effect to promote wound healing.

To overcome these shortcomings, in this study, we developed a solvent-free solubilization technique to obtain IB-loaded nanoparticles (IB@NPs) with high and stable antioxidant activity for efficient wound healing [28,29] (Figure 1). If IB is dispersed in an aqueous solution using solubilization technology, its encapsulation efficiency, long-term stability, and bioavailability can be increased, which can be applied in various fields. We identified the most optimized amphiphilic surfactants for IB and established formulations that could improve the encapsulation efficiency without the use of organic solvents during the encapsulation process. To confirm the encapsulation stability of IB, various physicochemical properties, including particle size, polydispersity index (PDI), surface charge, and morphology, were measured using dynamic light scattering (DLS) and staining tests. The antioxidant activity of IB@NPs was then evaluated using the 2,2-diphenyl-1-picrylhydrazyl (DPPH) assay and compared with that of unloaded IB. Furthermore, we evaluated the effect of IB@NPs on oxidative damage and ROS production in mouse NIH 3T3 fibroblasts. Moreover, we confirmed the biocompatibility and wound-healing abilities of IB@NPs using the in vitro cell model. Overall, these findings demonstrate the potential of IB@NPs as an antioxidant system for the treatment of various oxidative-stress-related diseases.

## 2. Materials and Methods

### 2.1. Materials

IB was purchased from Tokyo Chemical Industry Co. (Tokyo, Japan). Methylene blue, DPPH, and polyethylene glycol-40 hydrogenated castor oil (PEG-40 HCO) were purchased from Sigma Aldrich (St. Louis, MO, USA). Deionized water (DIW) and phosphate-buffered saline (PBS; pH 7.4) were purchased from HyClone (Logan, UT, USA). H_2_O_2_ (30%) was obtained from Junsei Chemical Co. (Tokyo, Japan). For the in vitro cell culture, Dulbecco’s modified Eagle’s medium (DMEM), penicillin–streptomycin, and fetal bovine serum (FBS) were obtained from Gibco (Grand Island, NY, USA). As reagents used for in vitro experiments, dimethyl sulfoxide-d6 (DMSO-d6, 99.8%) was purchased from Sigma-Aldrich, and 3-(4,5-dimethylthiazol-2-yl)-2,5-diphenyltetrazolium bromide (MTT) and 2,7-dichlorodihydrofluorescein diacetate (H2DCFDA) were purchased from Invitrogen (Carlsbad, CA, USA). All solvents were used as received without further purification.

### 2.2. Preparation of IB@NPs

IB@NPs were prepared via self-assembly in water after loading hydrophobic IB into an amphiphilic PEG-40 HCO consisting of a hydrophobic castor oil segment and a hydrophilic carboxylic acid segment. First, 0, 9, 15, or 30 mg of IB was added to 300 mg of PEG-40 HCO and then loaded at each concentration under magnetic stirring for 2 h at room temperature (25 °C). Second, using a syringe pump (LEGATO100, KD Scientific, Korea), IB containing PEG-40 HCO was added dropwise to 5 mL of DIW with stirring at 530 rpm. IB@NPs (loading content of 0, 3, 5, and 10 wt%) were stabilized by gentle stirring for 30 min. The hydrodynamic diameter, PDI, and zeta potential of IB@NPs were characterized by DLS with an electrophoretic light-scattering spectrophotometer (ELS-Z2, Otsuka Electronics Co., Tokyo, Japan). Finally, the unloaded IB was purified by ultrafiltration at 2000 rpm for 15 min using Amicon Ultra-15 centrifugal filters (molecular weight cut-off: 100 kDa). The loading content and the loading efficiency of the nanoparticles were characterized by high-performance liquid chromatography of unloaded IB [30] and then calculated using the following equations [31]:

$$Loading\;content\;\left(\%\right)=\left[\frac{\left(weight\;of\;fed\;IB-weight\;of\;unloaded\;IB\right)}{weight\;of\;NPs}\right]\times 100$$

$$Loading\;efficiency\;\left(\%\right)=\left[\frac{\left(weight\;of\;fed\;IB-weight\;of\;unloaded\;IB\right)}{weight\;of\;fed\;IB}\right]\times 100$$

### 2.3. Methylene Blue Staining of IB@NPs 

To visually verify the transparency and stability of the dispersed phase of the prepared composition, a dye solubility experiment was conducted using the water-soluble dye, methylene blue. First, methylene blue was diluted to 60 nM using DIW. Next, 10 mL of diluted methylene blue solution was added to 10 mL of IB@NPs and dispersed well. Finally, the initial state and the state after 30 days were visually observed to confirm the precipitation or dispersion of the nanoparticles.

### 2.4. Evaluation of the Stability of IB@NPs

The stability of IB@NPs was evaluated by DLS for 28 days. The hydrodynamic diameter and PDI of IB@NPs were determined at concentrations ranging from 0 to 10 wt% in an aqueous solution (DIW) at each time point: 0, 1, 3, 7, 14, and 28 days. The redispersion stability of the IB@NPs was evaluated after 3 days of lyophilization and resuspension in biological buffer (PBS, pH 7.4) and DIW at 100 rpm and 37 °C, at a concentration of 5 wt%, without the addition of a cryoprotectants. 

### 2.5. Antioxidant Activity of IB@NPs by DPPH Assays 

The antioxidant activities of IB and IB@NPs were analyzed using a DPPH assay [32]. First, ascorbic acid (AA), IB, and IB@NPs were diluted with DIW or ethanol to obtain two concentrations (3 and 5 wt%). Second, a 0.2 mM DPPH solution was prepared in ethanol and stored in the dark at 4 °C. Then, 50 µL of the prepared DPPH solution was mixed with 150 µL of each sample solution. The control group was DPPH solution (50 µL) mixed with DIW (150 µL), which barely exhibited any antioxidant activity. AA was used as a positive control for comparison. All reaction mixtures were left in the dark at room temperature for 1 h, and the absorbance of the mixture was measured at a wavelength of 515 nm using a microplate reader (VICTOR X5, PerkinElmer, Singapore, Republic of Singapore). Antioxidant activity was calculated using the following equation [33,34]:

$$Antioxidant\;activity\;\left(\%\right)=\left[\frac{\Delta A_{515}\;of\;control-\Delta A_{515}\;of\;sample}{\Delta A_{515}\;of\;control}\right]\times 100$$

### 2.6. In Vitro Cytotoxicity of IB@NPs

The cytotoxicity evaluation of IB@NPs was conducted using mouse embryonic fibroblast cells (NIH 3T3) that were cultured in DMEM containing 10% FBS and 1% penicillin–streptomycin. Cell viability measurements were conducted 24 h after exposure to IB@NPs using the MTT assay. Cells were seeded in 96-well plates at a density of 10,000 cells per well. After 24 h of incubation, the cells were treated with various concentrations of IB@NPs (250 nM to 5 µM) and incubated for 24 h at 37 °C [35,36]. Subsequently, the medium was replaced with MTT solution at a concentration of 1 mg/mL, fresh medium was added to each well, and incubation continued for 3 h. The medium was then removed, and DMSO-d6 was added to dissolve the purple formazan dye crystals. The absorbance of the formazan produced by viable cells was measured using a microplate reader (BioTek, Winooski, VT, USA) at 570 nm. Cell viability was calculated as a percentage using the following equation. All experiments were conducted in triplicate [37].

$$\mathrm{Cell}\;\mathrm{viability}\;\left(\%\right)=\left(\frac{\Delta A_{570}of\;test\;group}{\Delta A_{570}of\;control\;group}\right)\times 100$$

### 2.7. In Vitro Antioxidant Activity of IB@NPs

To evaluate the intracellular antioxidant activity of IB@NPs, NIH 3T3 fibroblasts were seeded in 96-well plates (10,000 cells/well) and cultured in an incubator for 24 h. ROS were generated in NIH 3T3 cells by stimulation with the oxidative stress agent H_2_O_2_. After treatment, changes in ROS levels were evaluated. First, different concentrations (50–500 nM) of IB@NPs and 5 μM H_2_O_2_ were added to NIH 3T3 cells and then incubated for 8 h. A negative control without H_2_O_2_ was used for comparison. After washing with PBS to remove the remaining medium, 10 μM of an ROS fluorescent indicator, H2DCFDA solution, was added to the NIH 3T3 cells and incubated for an additional 90 min in the dark. The in vitro antioxidant activity of IB@NPs was calculated by detecting the fluorescence intensity from dichlorofluorescein oxidized by ROS with an excitation wavelength of 485 nm and an emission wavelength of 535 nm using a microplate reader [13,14].

### 2.8. In Vitro Wound-Healing Activity of the IB@NPs

A scratch wound-healing assay is a common method for assessing cell proliferation and migration. First, NIH 3T3 fibroblasts were seeded at 150,000 cells per well into 24-well plates and grown for 24 h. After the NIH 3T3 cells adhered to the plate, the cells were scraped using a sterile P1000 micropipette tip to create a scratch wound. The cells were washed twice with DMEM to remove cell debris and then treated with 500 nM of IB@NPs. The control group received DMEM and penicillin–streptomycin without FBS. Cell wound closure was evaluated during incubation at 37 °C for 72 h at various time intervals (0, 6, 18, 24, 48, 72 h) using a microscope (KI-400, Korea Lab Tech, Korea). Cell wound gap distances were calculated using ImageJ software 1.8.0 (National Institutes of Health, Bethesda, MD, USA) [38].

### 2.9. Statistical Analysis

All experiments were performed in triplicate. The resulting data are shown as the mean ± standard deviation. Differences between experimental groups were compared using Student’s *t*-tests. Statistical significance for all evaluations was established as *p* < 0.05.

## 3. Results and Discussion

### 3.1. Characterization of IB@NPs

**Figure 2:**
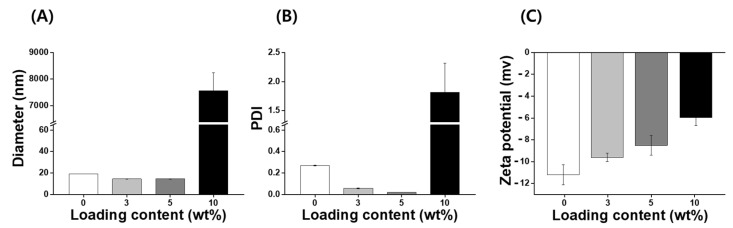
(**A**) Hydrodynamic diameters, (**B**) polydispersity index (PDI), and (**C**) zeta potential of IB@NPs with a loading content ranging from 0 to 10 wt%.

IB, a synthetic, short-chain benzoquinone, is a lipophilic antioxidant [19]. However, IB has low aqueous solubility and stability, which limits the use of pharmaceuticals or cosmetics. A number of different encapsulation materials have been used to improve the solubility, stability, and bioavailability of IB to increase their utilization in biotechnology [14,22,26]; however, nanocarriers for IB investigated to date require additional improvements with regard to the loading capacity and stability. Consequently, it is necessary to develop an improved IB carrier that can increase stabilization and antioxidant activity. In this study, nanoparticles with various concentrations of IB (ranging from 0 to 10 wt%) were prepared using an encapsulation method without the use of organic solvents such as ethanol, tetrahydrofuran, and chloroform. IB@NPs are stable nanoaggregates that self-assemble in an aqueous solution with a hydrophilic shell and hydrophobic core (Figure 1). The physicochemical properties (size, PDI, and zeta potential) of the IB@NPs were not affected with an increase in the IB content from 0 to 5 wt%. The hydrodynamic diameter of 5 wt% IB@NPs was 14.4 ± 0.12 nm (Figure 2A). However, IB@NPs with a loading content of 10 wt% had larger diameters of 7556 ± 689 nm, indicating that the IB was overloaded. The 5 wt% IB@NPs were well dispersed with PDI values of 0.02 ± 0.01 and a narrow particle size distribution (Figure 2B). The surface of the 5 wt% IB@NPs had a negative charge with a mean zeta potential of approximately −8.5 ± 0.89 mV. However, the surface charges of the IB@NPs became slightly less negative, ranging from –11 to –6 mV, when increasing the content of IB loaded (Figure 2C). High-performance liquid chromatography showed that the loading content and loading efficiency were 2.74 and 91.48, respectively, for the 3 wt% IB@NPs; 4.4 and 88.6, respectively, for the 5 wt% IB@NPs; and 5.33 and 53.3, respectively, for the 10 wt% IB@NPs.

### 3.2. Emulsification Stability of the IB@NPs

**Figure 3:**
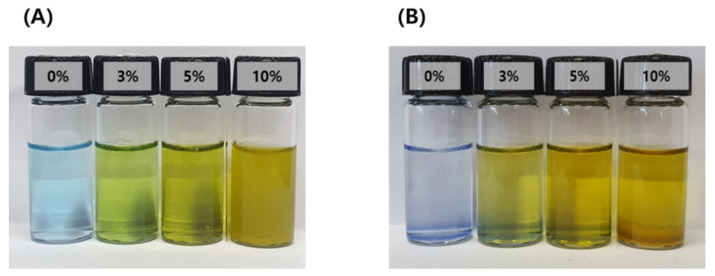
Methylene blue staining test of IB@NPs for confirmation of the stability of the microemulsion according to dye solubility. (**A**) Initial state of IB@NPs, (**B**) IB@NPs after 30 days.

Particles formed by micro-emulsification of the oil phase in the aqueous phase are thermodynamically unstable because of their high energy level. In other words, the system is metastable. Therefore, droplets dispersed by surfactants easily undergo phase changes through destabilization processes such as Ostwald ripening, flocculation, coalescence, creaming, and precipitation. The emulsification stability of the IB@NPs was confirmed by staining with water-soluble methylene blue to check the dispersion of the particles. Except for the 10 wt% IB@NPs, the other solutions were transparent in all compositions, and the particles were uniformly dispersed (Figure 3). The 10 wt% solution sedimented immediately upon preparation, which was attributed to the lack of a solubilizer capable of loading hydrophobic IB; thus, instability was confirmed by sedimentation of the 10 wt% solution. After 30 days, the 0 wt%, 3 wt%, and 5 wt% IB@NPs remained stable and transparent, suggesting that 5 wt% IB was the optimal loading content.

### 3.3. Long-Term and Redispersed Stability of the IB@NPs

**Figure 4:**
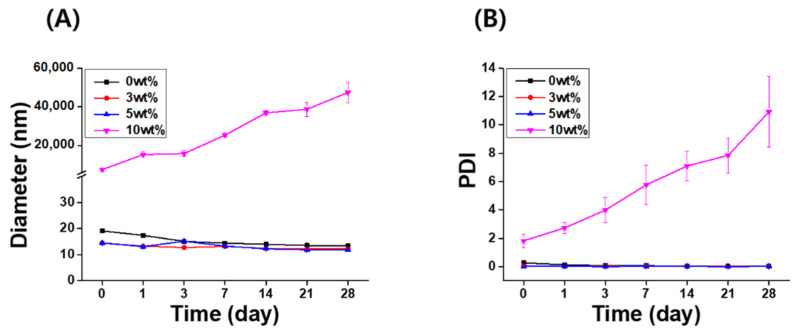
Long-term stability of IB@NPs at concentrations ranging from 0 to 10 wt%. Change in (**A**) hydrodynamic diameters and (**B**) the polydispersity index (PDI) of IB@NPs for 28 days.

**Figure 5:**
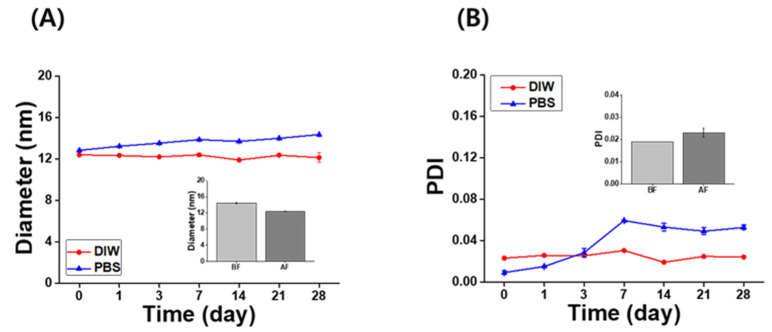
Long-term stability of the (**A**) hydrodynamic diameter and (**B**) polydispersity index (PDI) of 5 wt% IB@NPs redispersed in distilled water (DIW) and phosphate-buffered saline (PBS, pH 7.4) after lyophilization without any cryoprotectants for 28 days. Inset graphs are the redispersion stability of 5 wt% IB@NPs before freeze-drying (BF) and after freeze-drying (AF) in DIW.

The long-term stability of the IB@NPs was assessed under the following three conditions by observing the changes in nanoparticle size and PDI using DLS: (1) aqueous solution (DIW, 25 °C), (2) redispersed type after lyophilization in DIW, and (3) redispersed type after lyophilization in biological buffer solution (PBS, 37 °C, 100 rpm). In DIW, the initial size (Figure 4A) and PDI (Figure 4B) of IB@NPs were maintained for 28 days, with the exception of the 10 wt% solution. Moreover, the 5 wt% IB@NPs were successfully lyophilized and easily resuspended in DIW and PBS without any critical changes in their characteristics (i.e., diameter, Figure 5A; PDI, Figure 5B), facilitating ease of usage and storage. More importantly, to enhance dispersion, the stability of the IB@NPs was maintained without the addition of the cryoprotectants such as trehalose, glucose, or sucrose. These results suggest that our prepared IB@NPs could serve as an effective platform for stable drug delivery systems, as they show outstanding stability under different conditions, including redispersion.

### 3.4. DPPH Radical Scavenging Activities of the IB@NPs

**Figure 6:**
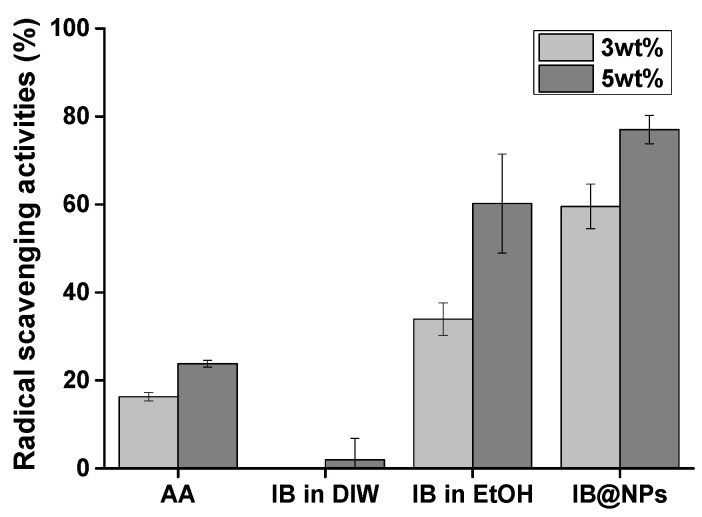
Antioxidant activity of IB@NPs compared with that of IB in distilled water (DIW), ethanol (EtOH), and ascorbic acid (AA), ranging from 3 wt% to 5 wt%, evaluated using the 2,2-diphenyl-1-picrylhydrazyl (DPPH) radical-scavenging assay.

IB is a stronger antioxidant than coenzyme Q10 [25]. However, it has lipophilic characteristics, making it difficult to optimize its antioxidant activity in an aqueous solution. For this reason, we developed and optimized IB@NPs based on previous studies to maximize IB activity in a water-soluble formation. First, the antioxidant activity of IB@NPs was compared with that of IB in ethanol and DIW using a DPPH radical scavenging assay (Figure 6). DPPH is an organic nitrogen radical with visible–ultraviolet absorption characteristics at 515 nm. When an antioxidant reacts with a solution of DPPH radicals, DPPH receives hydrogen atoms or electrons from the radical scavenger, the absorbance at 515 nm decreases, and the resulting solution changes color from violet to yellow. Therefore, the percentage of scavenged DPPH radicals was calculated using ultraviolet–visible measurements at a wavelength of 515 nm. IB was less effective in scavenging DPPH radicals than the positive control AA, as it is insoluble in an aqueous environment. Interestingly, IB, which is soluble in ethanol, was more effective at scavenging DPPH radicals than the positive control AA but was not as effective as IB@NPs. We consider this effect to be due to a decrease in the antioxidant activity of IB, even when dissolved in ethanol, from exposure to the external environment during the experiment, even for a brief period of time. Accordingly, the increase in the antioxidant activity of IB@NPs compared with that of the ethanol group is attributed to the effect of the nanoparticles on increasing the stability of IB and thus maintaining its antioxidant activity during encapsulation. In particular, the IB@NPs showed stronger antioxidant efficacy and free radical-scavenging activity as the concentration of IB increased. This confirmed that encapsulated IB has increased stability compared with that of externally exposed IB, resulting in increased radical-scavenging ability. Overall, these results suggest that the encapsulation of IB is important for its antioxidant activity.

### 3.5. In Vitro Cytotoxicity and Antioxidant Activity of IB@NPs

**Figure 7:**
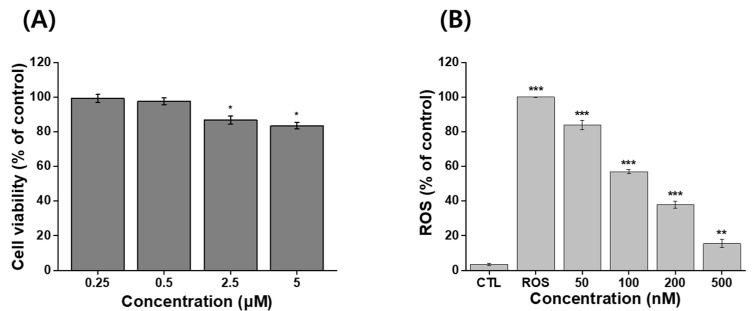
In vitro assays using MTT and H2DCFDA assay kits. (**A**) Cytotoxicity analysis of 5 wt% IB@NPs ranging from 250 nM to 5 μM. (**B**) Antioxidant activity of 5 wt% IB@NPs ranging from 50 nM to 500 nM; the control (CTL) and reactive oxygen species (ROS) groups represent the lowest and highest levels of ROS, respectively (* *p* < 0.05, ** *p* < 0.01, *** *p* < 0.005).

An MTT assay was used to evaluate the viability of NIH 3T3 cells following treatment with IB@NPs. There was a reduction in viability, but it was still acceptable given that more than 70% of the cells were viable. No toxic effects were observed in NIH 3T3 cells treated with the sample up to an IB concentration of 500 nM, as the viability of the cells was greater than 90 % at 24 h (Figure 7A). This indicated that IB@NPs are biocompatible up to a concentration of 500 nM and did not cause harmful effects. The IB@NPs exhibited significantly higher antioxidant activity at concentrations of 500 nM (Figure 7B). In vitro levels of ROS were quantified by analyzing H2DCFDA fluorescence after treatment with the oxidative stress agent H_2_O_2_. The levels were normalized to the control NIH 3T3 cells treated with H_2_O_2_ alone, set to 100%. As expected, exposure to IB@NPs effectively reduced the H_2_O_2_-induced increase in the ROS levels of NIH 3T3 cells. Furthermore, as the sample treatment concentration increased from 50 to 500 nM, the ROS level of IB@NPs significantly decreased to approximately 15.5%. This result demonstrated that the IB@NPs possessed very high antioxidant activity both in situ and in vitro.

### 3.6. In Vitro Wound-Healing Activity of IB@NPs 

**Figure 8:**
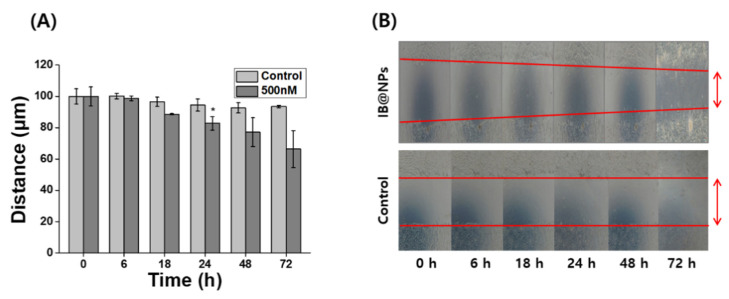
In vitro wound-healing efficacy of 5 wt% IB@NPs. (**A**) Wound closure of NIH 3T3 fibroblasts after treatment of IB@NPs for 3 days. (**B**) Microscopic photographs of wound-healing activity of IB@NPs (* *p* < 0.05).

Severely injured cells have high levels of ROS that delay wound repair, resulting in the inhibition of cell proliferation and migration [3]. Therefore, detoxification with ROS-scavenging substances can induce skin wound healing. In particular, scavenging activity and biological signaling induced by IB may play an important role in wound healing. Scratch assays are generally accepted as inexpensive and direct in vitro methods for observing wound healing, thus serving as an alternative to animal testing. After forming a scratch on the cell monolayer, the cells around the wound attempt to recover the damaged area through cell migration and proliferation. The wound-healing properties of IB@NPs were evaluated via scratch assays using a cell monolayer. After 24 h, the scratch in the untreated cells remained unchanged, whereas the wound length in the cells treated with IB@NPs decreased from 100 µm to 90 µm (Figure 8). The wound-healing effect increased over time after exposure to IB@NPs. After 72 h of treatment, the wound area reduced and was significantly smaller than the initial wound gap (*p* < 0.01). Thus, the IB@NPs treated to the wound site were able to promote cell migration and proliferation, accelerate the wound-healing activity of the cells, and inhibit tissue damage due to their outstanding ROS-scavenging activity.

## 4. Conclusions

In this study, IB@NPs were successfully developed using an emulsification method without organic solvents. The stability of the optimized IB@NPs was excellent. The IB@NPs were confirmed to be thermodynamically stable under redispersion conditions and biological buffer conditions. In addition, the IB@NPs were sufficiently stable for a long period and readily lyophilized to the powder state without the addition of cryoprotectants such as sucrose, glucose, or trehalose, resulting in improved dispersion. This suggests their feasibility of storage and transport for various applications. Furthermore, the improved aqueous solubility of IB@NPs significantly improves the bioavailability of IB. Consequently, the IB@NPs could effectively remove ROS and promote the wound healing of fibroblast cells without causing any cytotoxicity. In this respect, as a drug carrier with excellent stability, IB@NPs can be used in cosmeceutical applications.

## Data Availability

Not applicable.
